# Case Report: Late Successful Thrombectomy for Ischemic Stroke in a 2-Year-Old Child

**DOI:** 10.3389/fneur.2021.670565

**Published:** 2021-05-28

**Authors:** Nathalie Nasr, Louis Delamarre, Emmanuel Cheuret, Gerald Chausseray, Jean Marc Olivot, Philippe Acar, Fabrice Bonneville

**Affiliations:** ^1^Department of Neurology, Toulouse University Hospital, Université Toulouse III, INSERM UMR 1048, Toulouse, France; ^2^Department of Anesthesiology and Intensive Care, Toulouse University Hospital, Toulouse, France; ^3^Department of Pediatry, Pediatric Neurology Unit, Toulouse University Hospital, Toulouse, France; ^4^Department of Neurology, Toulouse University Hospital, Université Toulouse III, Toulouse, France; ^5^Department of Pediatry, Pediatric Cardiology Unit, Toulouse University Hospital, Université Toulouse III, Toulouse, France; ^6^Department of Neuroradiology, Toulouse University Hospital, Université Toulouse III, Toulouse, France

**Keywords:** thrombectomy, child, congenital heart disease, ischemic stroke, acute stroke

## Abstract

Despite extensive evidence of benefit of thrombectomy in adult ischemic stroke due to large-vessel occlusion in the 6-h window, its role remains uncertain in very young children. We describe hereafter the case of a 2-year-old female child who had a successful thrombectomy 9 h after stroke onset. The patient presented with right hemiplegia, central facial palsy, a normal level of consciousness, and speech difficulties. The PedNIHS score was 11. CT scan without contrast injection displayed spontaneous hyperdensity of the middle cerebral artery (MCA), with only limited early signs of ischemia (ASPECTS 8). CT angiography demonstrated occlusion of the proximal MCA with good collaterals. Thrombectomy was realized. Complete recanalization (TICI 3) was obtained under general anesthesia after two passes of a stent retriever. Time from symptoms onset to full recanalization was 9 h. The acute ischemic stroke was caused by embolic thrombus from a congenital heart disease. Clinical recovery was complete. Three months after the thrombectomy, the young patient was doing well without any neurological sequelae (PedNIHSS 0; modified Rankin Scale: 0). This case report is an example of a decision-making process to perform thrombectomy in a very young child, which included cardio-embolic etiology as a parameter that potentially might have participated to the successful outcome of the therapeutic procedure.

## Introduction

The role of thrombectomy in very young children for acute ischemic stroke due to proximal occlusion of middle cerebral artery (MCA) or internal carotid artery remains uncertain. An institutional local multidisciplinary consensus published that thrombectomy could be considered only in children older than 4 (1). Another challenge for treating these children is that they often present with a long delay from stroke onset to the recognition of stroke signs and subsequent transfer to a comprehensive stroke unit. This usually adds a second exclusion criteria based on delay for thrombectomy in children with stroke.

Our understanding of thrombectomy in this population is however changing, as recent retrospective data (2) have shown that thrombectomy in children is associated with very good outcome. The findings of another large multicentric retrospective study on thrombectomy for acute ischemic stroke in 73 patients aged <18 from 27 centers (The Save ChildS Study) suggested that neurological outcomes of the children were mostly favorable and comparable with those noted in adult trials (3). As for delay, two randomized trials in adults (DAWN and DEFUSE 3) demonstrated that the delay from stroke onset to thrombectomy can be extended beyond the usual 6-h window in patients who present a mismatch between the severity of the clinical deficit and the infarct volume, or between the infarct volume and the perfusion deficit (4, 5). Also, a secondary analysis of the Save Child study, which focused on thrombectomy performed between 6 and 24 h based on the presence of a mismatch between clinical deficit and infarct in 20 patients aged <18, revealed a good functional outcome in these patients (6).

We describe hereafter the case of a 2-year-old female child who had a successful thrombectomy performed 3 h beyond the 6-h window, for an acute ischemic stroke due to MCA occlusion caused by a thrombus originating from a congenital heart disease. The young girl had complete recovery.

## Patient Presentation, Initial Diagnosis, and Outcome

This child suffered from right hemiplegia at 7 p.m. Urgent medical attention was sought 3 h later, and the patient was oriented to the emergency ward of the nearby general hospital where a stroke unit for adults is available. CT scan without contrast injection (not shown) was performed at 11:00 p.m., and displayed spontaneous hyperdensity of the MCA, with only limited early signs of ischemia in the basal ganglia, localized in the deep left MCA territory, scored 8 on the Alberta Stroke Program Early CT Score (ASPECT score).

**Figure 1:**
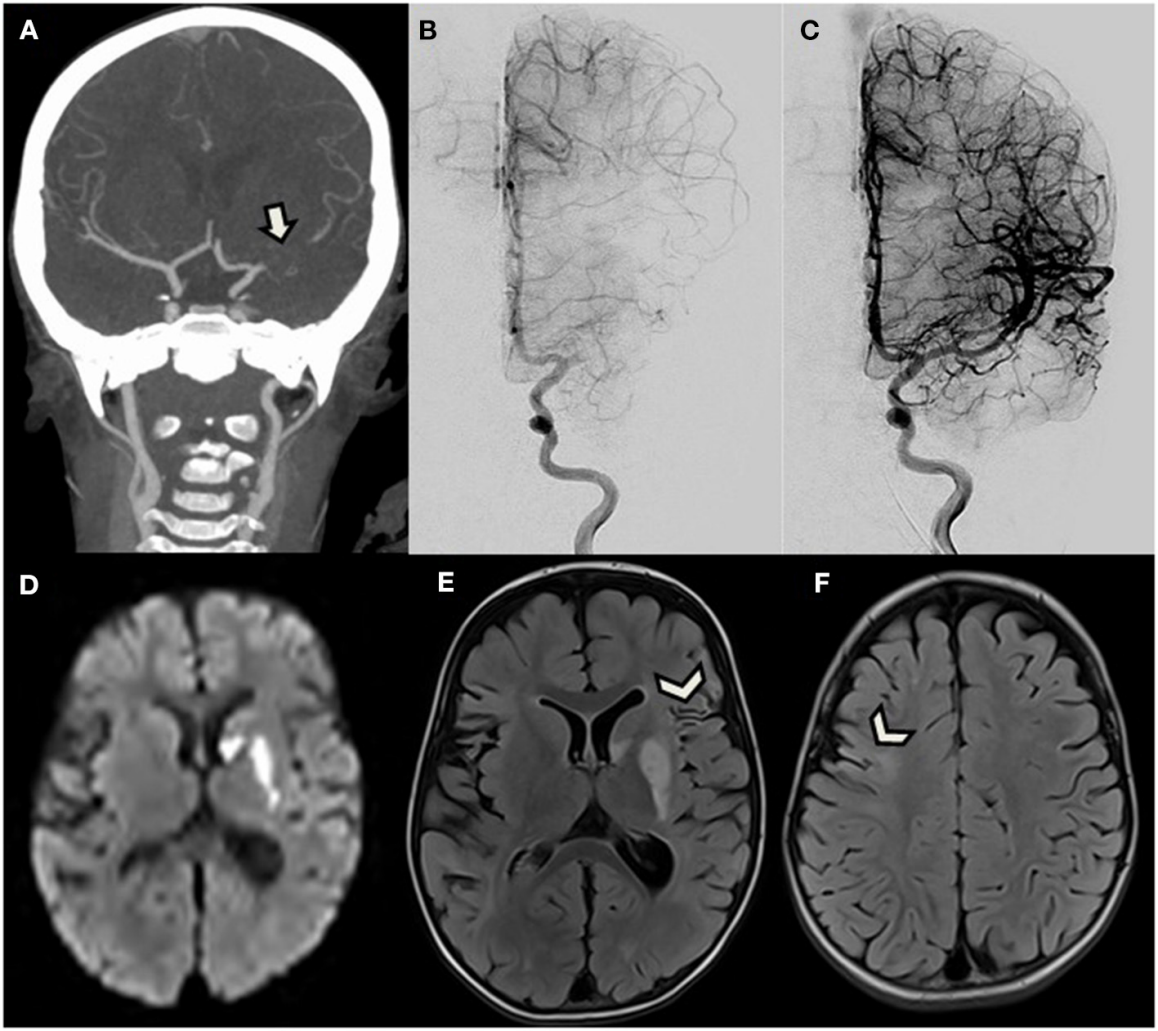
A 2.5-year-old girl with right hemiplegia. **(A)** Reformatted coronal CT angiogram shows proximal left middle cerebral artery occlusion (arrow) with good collateral flow. **(B,C)** Antero-posterior view of digital subtracted angiogram initially confirms CTA findings, and then demonstrates complete reopening after successful thrombectomy. **(D–F)** Brain MRI performed the following day. **(D)** Axial diffusion-weighted image and **(E,F)** FLAIR demonstrate acute ischemic stroke only limited to the basal ganglia, corresponding to the same areas with early ischemic signs on initial CT (not shown), without further extension. Images also reveal sequelae of ancient cortical ischemic lesions scattered in both middle cerebral artery territories (arrowheads).

The child was transferred to the tertiary-care university hospital stroke unit in order to consider thrombectomy. At admission, the patient presented with right hemiplegia, central facial palsy, a normal level of consciousness, and speech difficulties. She was playing with her doll with her left arm only. The Pediatric National Institutes of Health Stroke Scale (PedNIHSS) score was 11. CT angiography demonstrated occlusion of the proximal MCA and good collaterals beyond a 15-mm-long filling defect, taking over the cortical territory (Figure 1A).

Because of this favorable pattern with high ASPECT score and good collateral flow, and despite the time elapsed from symptoms onset, the patient was directly transferred to the angio suite where a pediatric cardiovascular anesthesiologist performed general anesthesia. A 4-F sheath was then introduced in the right common femoral artery. A 4-F catheter was navigated into the left internal carotid artery (ICA) over a 0.035-inch Terumo wire (Terumo Medical Corporation, Somerset, NJ). A first angiogram confirmed the occluded proximal M1 segment (Figure 1B). An Echelon 10 microcatheter (Medtronic) was navigated into the left MCA over a Traxcess 14 micro-guidewire (Terumo Medical Corporation, Somerset, NJ) and was carefully advanced through the thrombus occluding the M1 segment. A 3 × 23 mm MindFrame Capture LP stent retriever (Medtronic Inc. Minneapolis, MN) was then deployed into the occluded artery. After 4 min, the stent retriever and microcatheter were withdrawn together, while manual aspiration was performed from the 4-F guide catheter in the ICA, using a 20-cc syringe. A small amount of dark clot was trapped into the stent. Control angiogram then revealed occlusion of the terminal ICA. Because the new site of occlusion was more proximal, we considered that dislocated clot was unlikely responsible for the new occlusion and expected vasospasm to have rapidly developed after stent retrieval in this very young patient. Because vasospasm was thus suspected, 0.3 mg of nimodipine half-diluted with serum was manually injected in the 4-F catheter over 5 min. Subsequent angiogram confirmed vasospasm diagnosis, efficiently treated by nimodipine, by showing reopening of the ICA, and partial recanalization of the MCA bifurcation. A second pass of the strentriever was realized using the same technique, and the rest of the clot successfully withdrawn. Final angiogram demonstrated total reopening of the ICA, MCA, and ACA branches, ranked 3 on the Treatment in Cerebral Infarction Score (TICI), meaning complete recanalization (Figure 1C). The puncture to total recanalization time, as demonstrated by a TICI 3 score on the last run of DSA, was 43 min, and time from symptom onset to full recanalization was 9 h. Extubation was performed 45 min after the end of the procedure. One hour after thrombectomy, partial recovery of the right motor deficit was already noted. The next day, follow-up brain MRI revealed acute cerebral infarction only limited to areas with early ischemic signs on initial CT in the deep left MCA territory and revealed sequelae of older ischemic lesions in the territory of the right and left MCA (Figures 1D–F).

## Focus on Congenital Heart Disease That Caused Stroke

**Figure 2:**
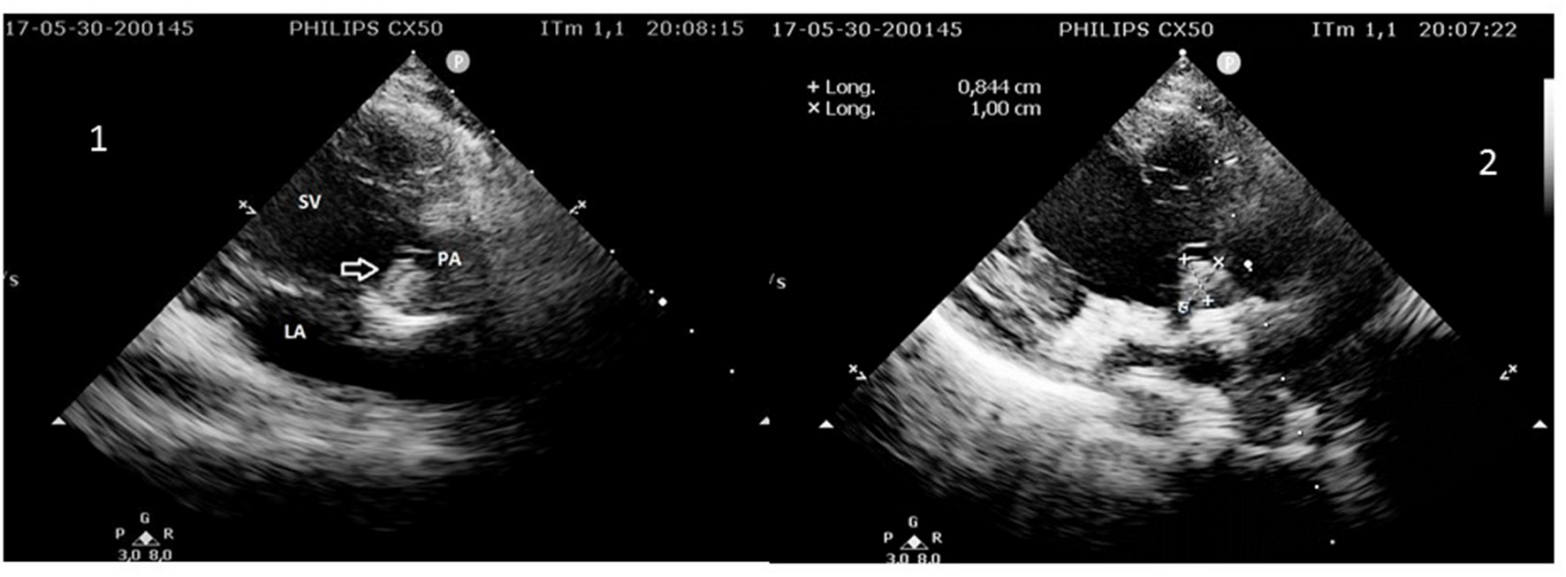
1. 2D echocardiography showing the mobile thrombus (arrow) inside the pulmonary artery (PA) trunk arising from the single ventricle (SV). LA, left atrium. 2. Measurement of the thrombus in the pulmonary artery (PA) trunk arising from the single ventricle (SV).

The child was known for complex congenital heart disease. Diagnosis of single ventricle had been established prenatally. Two palliative cardiac surgeries had been performed in neonate and at the age of 6 months. The pulmonary artery trunk had been sutured at the end of the second operation (partial cavopulmonary anastomosis). The thrombus that caused the stroke was located inside the pulmonary artery blind trunk (Figure 2). Plumonary artery and aorta arose from the same and single ventricle, which is the mechanism by which thrombi are believed to have embolized into the cerebral circulation.

After thrombectomy, the patient was treated with heparin and the intracardiac thrombus, monitored with cardiac echography, progressively disappeared. Clinical recovery was nearly complete, with only persistence of a subtle facial asymmetry, when the patient was discharged. Oral anticoagulant (coumadine) was then prescribed. Three months after the thrombectomy, the young patient was doing well without any neurological sequelae (PedNIHSS: 0; modified Rankin Scale: 0) with 85% pulse oximetry. She is under well-equilibrated anticoagulant treatment and is waiting for a third surgery planned to be a total cavopulmonary anastomosis which will be performed during the elementary school.

## Discussion

Recommendations concerning thrombectomy in children are scarce, because of lack of randomized trials in this population, especially at very young age. Only few years ago, some authors even advised against this treatment due to lack of sufficient data (7). More recently, a local multidisciplinary consensus recommended to propose thrombectomy only for pediatric patients older than 4 years (1). On the other hand, the prognosis of ischemic stroke in children is not less severe than in adults (8) with more than 70% morbidity (9). Thrombectomy is therefore recognized as an emerging option for selected children, even younger than 5 (10).

The multicentric retrospective study on thrombectomy for acute ischemic stroke in patients aged <18 published last year (The Save ChildS Study) included 73 patients from 27 centers with a median age of 11.3 years (interquartile range: 7–15). Its findings supported off-label thrombectomy in this population (3).

In the case we reported, the child was 2.5 years old and recanalization was achieved 9 h after stroke onset, beyond the recommended time window of 6 h commonly applied for adults who did not benefit from multimodal imaging assessing the ischemic penumbra (11). Still, clinical recovery was complete and the child had no adverse consequences of the treatment.

The outcome in this case report is in line with the findings of a secondary analysis of the Save ChildS Study that included 20 patients aged <18 who had thrombectomy between 6 and 24 h after stroke onset based on mismatch between clinical deficit and infarct (6). In the cited study by Sporns et al., the authors reported a higher proportion of good outcomes as compared to the DAWN and DEFUSE3 studies, and a similar proportion of patients with good outcome in the group treated > 6 h as compared to the group treated <6 h (6).

Of note, thrombectomy in our case report was performed under general anesthesia, which seems not to be associated with longer time to recanalization or with different outcome in adults (12).

Only two previous cases of thrombectomy in patients <4 years of age have been published in details. These two cases had successful outcomes and occurred as a consequence of cardioembolic strokes in 2- and 3-year-old boys (13, 14). Also, reviews on thrombectomy for ischemic stroke in children found that it was associated with a high recanalization rate and a very good clinical outcome (2, 15). The Save ChildS Study, which retrospectively included 73 patients under 18, found no vascular complications such as vessel rupture or dissection during the endovascular procedure. However, only seven patients with focal or bilateral cerebral arteriopathy were included. For the authors of the paper, an a priori selection bias may have played a role in excluding children with inflammatory cerebral arteriopathy from thrombectomy and may have participated to the observed low rate of hemorrhagic complications (3).

In our case, thrombectomy was achieved 9 h after stroke onset. Thrombolysis was not given because diagnosis was made more than 4.5 h after stroke onset. The criteria of the DAWN and Defuse 3 trials (4, 5), were they to be applied to a pediatric stroke, could not be implemented to our case. The volume of the infarct core and of the ischemic penumbra could not be assessed precisely, as we did not acquire MRI with DWI nor perfusion CT, indeed. However, the association of an ASPECT score of 8 with a proximal occlusion and good collateral flow demonstrated by CTA represents a favorable pattern for thrombectomy, as suggested by dedicated *post-hoc* analysis of the MR CLEAN trial (16).

In the case we describe, the cause of stroke was cardioembolic and was not due to a cerebral vasculopathy, such as moya-moya or transient cerebral arteriopathy, which is a more frequent cause of ischemic stroke in children (10, 17). In line with the few published cases of successful thrombectomy performed in cardioembolic strokes in children, we believe that the supposed cause of stroke should weigh more than age itself in the decision to perform thrombectomy in very young children with large-vessel occlusion. Presumed cardioembolic ischemic stroke with normal underlying cerebral vasculature may carry less risk for thrombectomy than stroke from cerebral arteriopathy.

## Conclusion

Thrombectomy is feasible in selected cases and may be clinically successful in very young children, even in case of relatively long delay between stroke onset and recanalization. The cardioembolic origin of the depicted stroke due to congenital heart disease, with absence of underlying vasculopathy, has potentially increased the odds of successful recanalization.

## Summary

This case report depicts late successful thrombectomy for ischemic stroke caused by congenital heart disease in a 2-year-old child, yielding complete recovery.

## Data Availability Statement

The raw data supporting the conclusions of this article will be made available by the authors, upon request.

## Ethics Statement

Ethical review and approval was not required for the study on human participants in accordance with the local legislation and institutional requirements. Written informed consent for the case-report to be published was provided by the participants' legal guardian/next of kin.

## Author Contributions

NN conceptualized the separation between ischemic stroke in small children caused by vasculopathy vs. ischemic stroke in small children caused by cardioembolic disease illustrated in this case-report, and its implication for thrombectomy, made substantial contribution to the acquisition, analysis and interpretation of data, and drafted and revised the manuscript. LD made substantial contribution to the acquisition, analysis and interpretation of data, depicted the anesthesia procedure, participated to depicting the congenital cardiopathy, and revised the manuscript critically for important intellectual content. EC made substantial contribution to the acquisition, analysis and interpretation of data, depicted the neurological recovery, and revised the manuscript critically for important intellectual content. GC made substantial contribution to the acquisition, analysis and interpretation of data, depicted the anesthesia procedure, and revised the manuscript critically for important intellectual content. JO made substantial contribution to the acquisition, analysis and interpretation of data, and revised the manuscript critically for important intellectual content. PA made substantial contribution to the acquisition, analysis and interpretation of data, depicted the congenital cardiopathy in this patient as well as the clinical follow up, and revised the manuscript critically for important intellectual content. FB made substantial contribution to the acquisition, analysis and interpretation of data, depicted the interventional procedure in this very small patient, participated to drafting the manuscript, and revised the manuscript critically for important intellectual content. All authors approved the final manuscript as submitted and agree to be accountable for all aspects of the work.

## Conflict of Interest

The authors declare that the research was conducted in the absence of any commercial or financial relationships that could be construed as a potential conflict of interest.

## References

[1] Buompadre MC Andres K Slater LA Mohseni-Bod H Guerguerian AM Branson H. Thrombectomy for acute stroke in childhood: a case report, literature review, and recommendations. Pediatr Neurol. (2017) 66:21–7. 10.1016/j.pediatrneurol.2016.09.007 27769730

[2] Satti S Chen J Sivapatham T Jayaraman M Orbach D. Mechanical thrombectomy for pediatric acute ischemic stroke: review of the literature. J Neurointerv Surg. (2017) 9:732–7. 10.1136/neurintsurg-2016-012320 27448827

[3] Sporns PB Sträter R Minnerup J Wiendl H Hanning U Chapot R. Feasibility, safety, and outcome of endovascular recanalization in childhood stroke: the save ChildS Study. JAMA Neurol. (2020) 77:25–34. 10.1001/jamaneurol.2019.3403 31609380 PMC6802048

[4] Nogueira RG Jadhav AP Haussen DC. Thrombectomy 6 to 24 hours after stroke with a mismatch between deficit and infarct. N Engl J Med. (2018) 378:11−21. 10.1056/NEJMoa1706442 29129157

[5] Albers GW Marks MP Kemp S. Thrombectomy for stroke at 6 to 16 hours with selection by perfusion imaging. N Engl J Med. (2018) 378:708–18. 10.1056/NEJMoa1713973 29364767 PMC6590673

[6] Sporns PB Psychogios MN Straeter R. Clinical diffusion mismatch to select pediatric patients for embolectomy 6 to 24 hours after stroke: an analysis of the Save ChildS Study. Neurology. (2021) 96:e343–51. 10.1212/WNL.0000000000011107 33144517 PMC7884981

[7] Ellis MJ Amlie-Lefond C Orbach DB. Endovascular therapy in children with acute ischemic stroke: review and recommendations. Neurology. (2012) 79:S158–64. 10.1212/WNL.0b013e31826958bf 23008391

[8] Bigi S Fischer U Wehrli E Mattle HP Boltshauser E Bürki S. Acute ischemic stroke in children versus young adults. Ann Neurol. (2011) 70:245–54. 10.1002/ana.22427 21823153

[9] Goldenberg NA1 Bernard TJ Fullerton HJ. International Pediatric Stroke Study Group. Antithrombotic treatments, outcomes, and prognostic factors in acute childhood-onset arterial ischaemic stroke: a multicentre, observational, cohort study. Lancet Neurol. (2009) 8:1120–7. 10.1016/S1474-4422(09)70241-8 19801204

[10] Sun LR Felling RJ Pearl MS. Endovascular mechanical thrombectomy for acute stroke in young children. J Neurointerv Surg. (2019) 11:554–58. 10.1136/neurintsurg-2018-014540 30842305

[11] Powers WJ Derdeyn CP Biller J Coffey CS Hoh BL Jauch EC. American Heart Association/American Stroke Association focused update of the 2013 guidelines for the early management of patients with acute ischemic stroke regarding endovascular treatment: a guideline for healthcare professionals from the American Heart Association/ American Stroke Association. Stroke. (2015) 46:3020–35. 10.1161/STR.0000000000000074 26123479

[12] Vukasinovic I Darcourt J Guenego A Michelozzi C Januel AC Bonneville F. Toulouse Stroke Group. Real life impact of anesthesia strategy for mechanical thrombectomy on the delay, recanalization and outcome in acute ischemic stroke patients. J Neuroradiol. (2019) 46:238–42. 10.1016/j.neurad.2018.09.005 30389509

[13] Stidd DA Lopes DK. Successful mechanical thrombectomy in a 2- year-old male through a 4-French guide catheter. Neurointervention. (2014) 9:94–100. 10.5469/neuroint.2014.9.2.94 25426305 PMC4239415

[14] Gerstl L Olivieri M Heinen F. Successful mechanical thrombectomy in a three-year-old boy with cardioembolic occlusion of both the basilar artery and the left middle cerebral artery. Eur J Paediatr Neurol. (2016) 20:962–5. 10.1016/j.ejpn.2016.07.014 27477566

[15] Bhatia K Kortman H Blair C. Mechanical thrombectomy in pediatric stroke: systematic review, individual patient data meta-analysis, and case series. J Neurosurg Pediatr. (2019) 9:1–14. 10.3171/2019.5.PEDS19126 31398697

[16] Berkhemer OA Jansen IG Beumer D Fransen PS Van Den Berg LA Yoo AJ. Collateral status on baseline computed tomographic angiography and intra-arterial treatment effect in patients with proximal anterior circulation stroke. Stroke. (2016) 47:768–76. 10.1161/STROKEAHA.115.011788 26903582

[17] Chabrier S Husson B Lasjaunias P Landrieu P Tardieu M. Stroke in childhood: outcome and recurrence risk by mechanism in 59 patients. J Child Neurol. (2000) 15:290–4. 10.1177/088307380001500504 10830194

